# Nitrogen and lysine utilization efficiencies, protein turnover, and blood urea concentrations in crossbred grower pigs at marginal dietary lysine concentration

**DOI:** 10.1093/jas/skad335

**Published:** 2023-09-29

**Authors:** Daniel Berghaus, Eva Haese, Ramona Weishaar, Naomi Sarpong, Alina Kurz, Jana Seifert, Amélia Camarinha-Silva, Jörn Bennewitz, Thilo Chillon, Volker Stefanski, Markus Rodehutscord

**Affiliations:** Institute of Animal Science, University of Hohenheim, Stuttgart, Germany; Institute of Animal Science, University of Hohenheim, Stuttgart, Germany; Institute of Animal Science, University of Hohenheim, Stuttgart, Germany; Institute of Animal Science, University of Hohenheim, Stuttgart, Germany; Institute of Animal Science, University of Hohenheim, Stuttgart, Germany; Institute of Animal Science, University of Hohenheim, Stuttgart, Germany; Institute of Animal Science, University of Hohenheim, Stuttgart, Germany; Institute of Animal Science, University of Hohenheim, Stuttgart, Germany; Institute of Animal Science, University of Hohenheim, Stuttgart, Germany

**Keywords:** lysine, nitrogen, protein turnover, swine, utilization, ^15^N glycine

## Abstract

Nitrogen utilization efficiency (**NUE**) and lysine utilization efficiency (**LUE**) are key indicators of sustainable pork production and vary depending on nutritional and non-nutritional factors. The objective was to study NUE and LUE together with concentrations of blood urea nitrogen (**BUN**) and other metabolites in growing pigs fed diets with marginal Lys concentrations at 11–13 wk (40.5 kg mean BW) and 14 to 16 wk (60.2 kg mean BW). The cereal grain–soybean meal-based diets contained 10.6 and 7.9 g Lys/kg DM in periods 1 and 2, respectively. Feed intake and BW were measured for 508 individually penned pigs, and blood samples were collected 5 h after morning feeding at weeks 13 and 16. A subgroup of 48 barrows was used in a nitrogen (**N**) metabolism trial at weeks 13 and 16. In this subgroup, the mean N retention of pigs (27.3 g N/d) and mean LUE (70%) were not different between the periods, but NUE was higher in period 1 (47%) than in period 2 (43%) (*P* < 0.001). After administration of a single dose of ^15^N labeled glycine and measurement of ^15^N recovery in urine, the calculated whole-body protein turnover did not differ between the periods. The rate of protein synthesis was positively correlated with NUE (*P* < 0.001), but protein degradation was not. Excretion of urea-N in urine accounted for 80% of the total urinary N and was positively correlated with BUN. The N retention of all 508 pigs was estimated using an equation that was derived from the N metabolism data. N retention was on average 31.4 g/d, equal in both periods, and higher in barrows than in gilts in period 2, but not in period 1 (*P* = 0.003). The calculated NUE was, on average, 47% and was lower in barrows than in gilts (*P* < 0.001) and higher in period 1 than in period 2 (*P* < 0.001). The calculated LUE was, on average, 71%, and was lower in barrows than in gilts in period 2, but not in period 1 (*P* < 0.001). The BUN concentration was higher in barrows than in gilts (*P* < 0.001) and higher in period 1 than in period 2 (*P* < 0.001). BUN concentration was negatively correlated with NUE in Periods 1 (r = -0.50) and 2 (r = -0.15) (*P* < 0.05). We concluded that the maximum LUE was in the range of 70–72% under the conditions of this study, and only small differences between the periods and sexes existed. Protein synthesis, rather than degradation, appears to affect NUE. BUN concentration may be useful for estimating NUE in a large group of animals fed a diet with a marginal Lys concentration.

## Teaser

The mean utilization of lysine by growing pigs can reach 72% when fed diets containing cereal grains and soybean meal. Nitrogen utilization varied among pigs and was associated with blood urea concentration and whole-body protein synthesis.

## INTRODUCTION

The increasing global protein demand, cost of feed protein, and negative effects on the environment arising from excess amino acid supply with feed and subsequent nitrogen (**N**) excretion necessitate research into an improved understanding of factors that influence N utilization efficiency (**NUE**; defined by N retention in the body relative to N intake) and how it can be increased in growing pigs. In their recent review of the literature, Shurson and Kerr (2023) concluded that pig feeding is most relevant for an improved sustainability of the pork production sector. Lysine utilization efficiency (**LUE**; Lys retention relative to Lys intake) is particularly relevant to NUE because Lys is the first limiting amino acid for protein retention in growing pigs under most feeding conditions. When Lys intake exceeds Lys requirement, LUE is decreased with an increased Lys intake. When Lys intake is marginally below the requirement, pigs may not achieve maximum protein retention, but show their potential maximum LUE and NUE under prevailing study conditions. The variations in LUE and NUE in a pig population studied at a marginal Lys supply is of interest, for instance, in the development of precision feeding and breeding of pigs.

Urea is the main end-product of metabolic amino acid catabolism and ammonia detoxification in the liver. Blood urea nitrogen (**BUN**) concentration is related to liver urea synthesis and urinary N excretion (Berschauer et al., 1983; Zervas and Zijlstra, 2002a). Correspondingly, BUN has been suggested to be an indicator of NUE (Berschauer et al., 1983; Chen et al., 1999). These studies used different concentrations of CP in their diets, thus stimulating differences in hepatic urea synthesis through different excess intake of amino acids. However, we are unaware of any studies that have tested BUN as an indicator of NUE in pigs fed a diet marginally deficient in Lys.

When the Lys supply to pigs is marginal, non-nutritional factors are likely to affect the variations in LUE and NUE in a group of pigs. Protein retention in the body results from differences in protein synthesis and simultaneous protein degradation. In pigs, whole-body protein synthesis is much higher than protein retention (Simon, 1989; Rivera-Ferre et al., 2006) and protein degradation contributes to amino acid catabolism, thereby reducing NUE and LUE. Pigs may synthesize proteins to different extents depending on their genetic potential and under the control of metabolic hormones such as insulin-like growth factor 1 (**IGF-1**) and cortisol (Sève and Ponter, 1997; Weiler et al., 1998). Therefore, the ratio of whole-body protein synthesis to degradation may differ between pigs and contribute to variations in NUE and LUE.

The objectives of this study were to investigate the variations in NUE and LUE in growing pigs at a marginal level of Lys intake and evaluate BUN as an indicator of NUE under such supply conditions. Another objective was to study whether whole-body protein turnover and related blood metabolites contribute to variations in NUE. Finally, we compared NUE and LUE of growing barrows and gilts. The hypothesis was that BUN is an easy-to-measure indicator of NUE in a group of pigs fed marginal Lys supply levels and that NUE may be affected by the ratio of whole-body protein synthesis to degradation.

## MATERIAL AND METHODS

All animal procedures were reviewed and approved by the Regierungspräsidium Tübingen, Germany (approval number 35/9185.81-4), according to German animal welfare regulations. The animal study was conducted at the University Agricultural Experiment Station ‘Unterer Lindenhof’ in Eningen unter Achalm, Germany, and lasted 30 months. This study comprised a feeding trial with 508 individually penned pigs. Of these, 48 barrows were additionally used in the concurrent N metabolism trial. Based on the results of the N metabolism trial and blood metabolites, the NUE was estimated for all 508 pigs in this study.

### Experimental diets

All pigs were fed two diets in two consecutive periods. The diets were based on wheat, barley, and soybean meal and calculated to meet or exceed the requirements of growing pigs at 40 and 70 kg BW (period 1 and 2, respectively), according to the Gesellschaft für Ernährungsphysiologie (GfE, 2008), except for Lys (Table 1). The concentration of standardized prececal digestible Lys was calculated at 0.60 g/MJ ME in period 1 and 0.45 g/MJ ME in period 2, which was 10% below the recommended concentration. The concentrations of other amino acids were higher than those recommended. Free amino acids and enzymes were not included in the diets. A new batch was mixed at the research station for each cohort of pigs entering the experiment, and each batch was analyzed. The mean analyzed concentrations of CP were 215 and 177 g/kg DM and those of Lys were 10.6 and 7.9 g/kg DM in periods 1 and 2, respectively (Table 1).

**Table 1.** Ingredient composition of the experimental diets

	Period	
	1	2
Ingredients, g/kg as fed		
Wheat	430	430
Barley	300	402
Soybean meal	240	140
Soybean oil	3	5
Mineral–vitamin mix^1^	27	23
Calculated concentrations		
ME, MJ/kg DM	14.9	14.8
CP, g/kg DM	230	190
Standardized prececal digestible Lys, g/kg DM	8.9	6.7
Analyzed concentrations^2^, g/kg DM		
CP (N × 6.25)	215 ± 6.1	177 ± 6.3
Lys	10.6 ± 0.5	7.9 ± 0.3
Met + Cys	7.0 ± 0.3	6.1 ± 0.3
Thr	8.0 ± 0.1	6.4 ± 0.2
Val	10.0 ± 0.4	8.3 ± 0.4
Leu	16.1 ± 0.6	13.0 ± 0.6
Ile	8.8 ± 0.4	6.9 ± 0.3
His	5.8 ± 0.2	4.7 ± 0.2
Phe + Tyr	17.7 ± 0.7	14.4 ± 0.7

^1^Provided the following per kilogram of diet in period 1: vitamin A, 6,000 IU; vitamin D, 1,400 IU; vitamin E, 110 IU as α-tocopherol acetate; vitamin K, 1.4 mg; niacin, 10.5 mg; pantothenic acid, 2.5 mg; folic acid, 0.8 mg; pyridoxine, 21 mg; thiamine, 2.8 mg; choline chloride, 340 mg; biotin, 0.22 mg; vitamin B_12_, 0.02 mg; Ca, 6.7 g; P, 1.4 g; Na, 1.4 g; Mg, 0.6 g; Zn, 53 mg as ZnO; Cu, 8 mg as CuSO_4_; Fe, 55 mg as FeSO_4_; Mn, 55 mg as MnO; I, 1.4 mg as Ca(IO_3_)_2_; and Se, 0.3 mg as Na_2_SeO_3_. The contribution in period 2 was lower according to the lower inclusion rate of the mineral–vitamin mix.

^2^Values are means of 21 (period 1) and 19 (Period 2) diet samples analyzed during the study ± SD.

### Animals and Procedures

Pigs were obtained from the herd at the research station. Over a period of 30 months, Landrace sows were mated by artificial insemination in a predefined scheme to 20 unrelated Piétrain boars kept at the reproduction station at Herbertingen, Germany, using a 2-week production rhythm. Male piglets were surgically castrated under analgesia (Metacam) and anesthesia (Ketamidor) during the first week of life. During rearing, the piglets were vaccinated against *Mycoplasma hyopneumoniae* (Stellamune One), *Lawsonia intracellularis* (Enterisol Ileitis), and porcine circovirus (Circo Flex). The piglets were weaned at 28 days of age and fed a standard piglet starter feed.

At 11 weeks of age and a mean BW of 29.5 kg, the piglets were transferred to the experimental barn. For each of the 21 cohorts of 25 piglets used over 30 months, four piglets per litter were selected from six litters of a farrowing group. From each litter, one female and one castrated male, each with the highest BW and those closest to the mean BW of the litter, were chosen. The 25^th^ piglet was randomly selected from each of the six litters. The pigs were individually penned; each pen was 2.7 m^2^ in size, thereof 0.5 m^2^ was slatted floor and the remainder of the concrete floor had some sawdust bedding. The pens were separated by metal grids with a 10 cm distance between bars to allow some animal contact. Each pen was equipped with a low-pressure nipple drinker and a height-adjustable feeding trough. Water was available continuously. Feed was provided at 1.5 ME requirement for an average daily gain of 950 g (GfE, 2008), adjusted weekly and representing ad libitum condition for most of the pigs. Feed was added to the trough from previously weighed buckets in the morning and afternoon and orts were removed and weighed every week. The pigs were weighed once a week.

Period 1 diet was provided to all pigs, starting with the move to the individual pens in week 11. At week 13, samples were collected as described below. From week 14 onward, period 2 diet was provided, and samples were collected in week 16. The experiment continued until a BW of ~90 kg was reached at the age of 21 wk, and the pigs were transferred to a commercial slaughterhouse. In weeks 13 and 16, the feed intake was quantified for each pig for five consecutive days. Fecal samples were collected for microbiota analysis to be reported elsewhere. On days 2, 3, and 4, blood samples were obtained 5 h after the morning feeding from the *vena ­jugularis* of each animal by puncturing with a 2-mm Strauss cannula. Blood was collected in silicate-carrying test tubes (Kabe Labortechnik GmbH, Nümbrecht, Germany) and centrifuged for 10 min at 1,000 × *g*, and the obtained serum was stored at −20 °C.

Of the 508 pigs, 48 were used in a concurrent N metabolism trial. Eight barrows from six cohorts were used to represent the study time span. The pigs were housed individually in stainless steel metabolic units (0.8 m × 1.5 m) on plastic-coated slatted floors. Feed was provided semi ad libitum from previously weighed buckets in two meals per day in such a way that pigs were fed close to satiation, but feed spilling was minimized. Spilled feed was collected from the tray underneath once observed. Low-pressure drinking nipples provided free access to water. After two days of adaptation to the metabolic unit, feces and urine were collected separately for four days. Feces were collected in plastic bags attached to the anus of the pig, weighed, stored at −20 °C and pooled after thawing, and homogenized. One sample was used for N analysis of the original feces, and another dried at 65 °C for other analyses. Urine was obtained from the stainless-steel trays underneath and collected in a bucket with 20% diluted sulfuric acid to maintain a pH value <2. The buckets were replaced when urination was observed or at least every 12 h, the content was weighed and stirred, 10% taken into a pooled sample, and stored in airtight bottles at 4 °C. After each period, pooled urine samples were homogenized in a 5-L beaker, and 30 mL was collected for subsequent analyses.

As part of the N metabolism trial, protein turnover was determined using the end-product method (Duggleby and Waterlow, 2005) after the oral administration of a single dose of ^15^N-labeled Gly (Rivera-Ferre et al., 2006). A subsample of the urine from the first 12-h collection interval was taken to measure the natural abundance of ^15^N of urinary N. Then, a gelatin capsule containing 6 mg ^15^N-labelled Gly/kg BW (99% ^15^N excess; Eurisotop, Saint Aubin Cedex, France), corresponding to 1.18 mg ^15^N/kg BW, was placed behind the base of the tongue to ensure immediate and complete swallowing of the marker. During the first two collection periods, a urine sample from each 12-h collection interval was collected to follow the course of ^15^N excretion in the urine and an exponential equation was fitted to the data (Supplementary Fig. S1). In all collection periods, a bulk urine sample from the 84-h period following ^15^N administration was obtained to measure ^15^N enrichment. More than 96% of the calculated plateau in ^15^N excretion was reached at 72 h after ^15^N application (Supplementary Fig. S1), and we concluded that analysis of the 84-h bulk sample was adequate to determine the proportion of the ^15^N dose excreted in urine. Blood samples were collected and processed as described previously.

### Sample processing and chemical analyses

The feed samples were ground through a 0.5-mm sieve (Ultra Centrifugal Mill ZM 200; Retsch GmbH, Haan, Germany) to determine DM (method 3.1) and Kjeldahl N (method 4.1.1, Verband Deutscher Landwirtschaftlicher Untersuchungs- und Forschungsanstalten (VDLUFA, 2007)). The CP concentration was calculated as N × 6.25. Feed and fecal samples were pulverized using a vibrating cup mill (Pulverisette 9; Fritsch GmbH, Idar-Oberstein, Germany) for the analysis of amino acids (feed only), as described by Rodehutscord et al. (2004), and TiO_2_ by inductively coupled optical emission spectrometry, as described by Boguhn et al. (2009). Total N in the feces was determined using samples collected directly after thawing, and the method was as described for feed. Thawed urine samples were analyzed for total N, as described previously. The urea concentration in urine was determined enzymatically using a urea/ammonia test kit (R-Biopharm AG, Darmstadt, Germany), and photometric extinction was measured at 340 nm (Evolution 201 UV-visible spectrophotometer; Thermo Fisher Scientific, Waltham, USA). The urea N concentration was calculated by applying a factor of 0.467. To determine ^15^N enrichment, 10 mL urine was neutralized with 1 M sodium hydroxide, and 0.8 g silica carrier was added and the mix freeze-dried using a delta 1 to 24 freeze dryer (Christ, Osterode am Harz, Germany). Dry material was weighed into tin capsules (IVA Analysentechnik, Meerbusch, Germany) using a precision scale (AD-4 Autobalance; PerkinElmer Inc., Waltham, MA, USA). For isotope ratio determination, an elemental analyser (EA 1108; Carlo Erba Instruments, Biberach, Germany) equipped with an autosampler (AS 200 LS), a ConFlo II open split interface (MS Finnigan MAT; Bremen, Germany), and an isotope ratio mass spectrometer (MS Finnigan MAT, Bremen, Germany) were used. Vacuum was generated by a Pfeiffer Duo 2.5 and a Pfeiffer TPH 062 pump (Pfeiffer Vacuum GmbH, Asslar, Germany). Helium 5.0 was used as carrier gas at ~100 mL/min, oxygen 5.0 was used for combustion at 10 to 15 mL/min, nitrogen 4.6 was used as servo gas at 350 kPa. Quartz reactors were used, the oxidation reactor was equipped with chromium oxide and silvered cobaltous oxide and held at 1,040 °C, the reduction reactor was equipped with copper and copper oxide and held at 650 °C. Samples were combusted with oxygen, C-containing substances were oxidized and subsequently N-containing substances were reduced to nitrogen. The gas stream passed a water trap filled with magnesium perchloride and in the next step separation of N_2_ and CO_2_ was performed on a Poropak PQS stainless steel column, 2 m at 41 °C. Via the Con Flow Interface, gases entered the mass spectrometer and the isotope ratio was determined. Calculations were done using Tracer MAT software, 5.34. Urea A and Urea B of the International Atomic Energy Acency (Vienna, Austria) were used as standards. Urinary creatinine was analyzed using a commercial enzyme reaction kit (553-886; mti-diagnostics GmbH, Idstein, Germany), as described by Wesoly et al. (2015). Cortisol in the urine was determined using a radioimmunological assay (Claus and Weiler, 1996) as described by Wesoly et al. (2015).

Blood serum was analyzed for urea by photometry (Beckman Coulter, Brea, USA) in a coupled enzyme reaction assay using urease to release ammonia and measurement of NADH following glutamate formation (IDEXX BioResearch Vet Met Labor GmbH, Kornwestheim, Germany). BUN was calculated using a factor of 0.467. For serum cortisol and IGF-1 determination, samples from three consecutive days were pooled into one sample and analyzed for cortisol using a radioimmunological assay described by Engert et al., (2017). IGF-1 was determined using a commercial automated chemiluminescent immunoassay with the IDS-iSYS automated system for human samples (Immunodiagnostic Systems Holdings Ltd., East Boldon, UK), as described previously (Bidlingmaier et al., 2014).

### Calculations

Nitrogen retention **(NR**) was calculated as the difference between daily N intake (**NI**) with feed and daily N excretion in feces and urine. NUE was calculated as follows:


$$\begin{array}{l} NUE(\;\%\;)=\;\frac{NR\;\;\;(g/d)}{NI\;\;\;(g/d)}\;\times 100 \end{array}$$ (1)

LUE was calculated assuming a Lys concentration in gained body protein of 7.2% (GfE, 2008), which is equivalent to 0.45 g Lys per 1 g retained N:


$$\begin{array}{l} LUE(\;\%\;)=\;\frac{NR(g/d)\times 0.45}{Lys\;\;\;intake(g/d)} \end{array}$$ (2)

where the daily Lys intake was calculated from the daily feed intake, and the analyzed Lys concentration of the feed.

A two-pool model was chosen to calculate body protein turnover based on the end-product method evaluated by Duggleby and Waterlow (2005). This calculation implies that, because all amino acids for different processes originate from the free amino acid pool of the blood, ^15^N of a single oral dose of a labeled amino acid is used in the same proportion for de novo protein synthesis and oxidation, with oxidation reflected by ^15^N excretion in urine. This also implies that ^15^N recycling from body proteins to the blood pool was irrelevant during the observation period.

The total N flux in the body protein pool was calculated as


$$\begin{array}{l} Q=\frac{d}{\varepsilon_{\left(t\right)}}\;with\;\varepsilon_{\left(t\right)}=\frac{e_{x}}{E_{x}} \end{array}$$ (3)

where *Q* is the N flux, *d* is the administered dose of ^15^N excess above natural abundance (^15^Nʹ) (mg), ε is the ^15^Nʹ in the end product (total urinary N) within the collection period *t*, *e*_*x*_ is the amount of tracer excreted in the end product (mg 15Nʹ/84 h), and *E*_*x*_ is the amount of total N excreted in the urine (g total N/84 h). Under steady-state conditions, protein synthesis and degradation can be determined using the following relationships:


$$\begin{array}{l} Q=S+E_{T}=D+I \end{array}$$ (4)

where *Q* is the body protein turnover or N flux (g N/d), *S* is the body protein synthesis (g N/d), *E*_T_ is the total urinary N excretion (g/d), *D* is the body protein degradation (g N/d), and I is the amount of dietary N entering the free amino acid pool (g/d) (Duggleby and Waterlow, 2005). The *I* value was calculated using measured N digestibility and intake values. The results were converted from N to protein using a factor of 6.25. Synthesis and degradation values were expressed in relation to the amount of body protein during the measurement period. The body protein content was calculated using Eq. 8 of GfE (2008) and an empty body weight of 94% of BW.

### Statistical analysis and regressions

Data of the N metabolism trial were analyzed using the MIXED procedure in SAS (version 9.4; SAS Institute Inc., Cary, NC, USA), with individual pigs as the experimental unit. Normal distribution and homogeneity of variance were tested prior to statistical analysis. If the results of the variables within a period were not normally distributed, the data were either square root, log-transformed, or logit-transformed for percentages. The statistical model was as follows:


$$\begin{array}{l} y_{ij}=\mu +\alpha_{i}+\beta_{j}+e_{ij} \end{array}$$ (5)

where *y*_*ij*_ is the dependent trait, µ is the overall mean, α_*i*_ is the fixed effect of *i*th period, β_*j*_ is the random effect of *j*th animal, and *e*_*ij*_ is the residual error. Differences between periods 1 and 2 were determined using pairwise *t* tests, and statistical significance was declared at *P* ≤ 0.05.

Multiple regression analysis was performed to predict NR based on performance data and blood metabolites using a macro for the MIXED procedure in SAS. The macro simultaneously compared all possible combinations of the considered variables and ranked the derived models based on the Akaike information criterion (**AIC**). The following model was used and adjusted for the inclusion of either linear or quadratic effects and interactions:


$$\begin{array}{l} \begin{array}{llll} & Y_{i}=a_{i}+\alpha_{i}+\;b_{1}\times V_{1}+b_{2}\times V_{2}+b_{3}\times (V_{1}\;\times V_{2})\; & +\;b_{4}\times V_{1}^{2}+b_{5}\times V_{2}^{2}+\ldots +\;b_{n-4}\;\times \;V_{i-1}+\;b_{n-3}\times V_{i}\; & +\;b_{n-2}\times (V_{i-1\;}\times V_{i})+b_{n-1}\times V_{i-1}^{2}+b_{n}\times V_{i}^{2}+e_{i}\; \end{array} \end{array}$$ (6)

where *Y*_*i*_ is the value of the response trait (NR), *a*_*i*_ is the intercept, α_*i*_ is the fixed effect of *i*th period, *b*_1−*n*_ is the regression coefficients of the respective variables, *V*_1−*i*_ is the estimation variables, and *e*_*i*_ is the residual error. Observations of the same animal in the two periods were considered repeated measures. The significance of the derived regression coefficients was validated using bootstrapping to avoid overfitting. One thousand additional same-sized datasets were created from the initial dataset through multiple resampling by using the SURVEYSELECT procedure in SAS. Using these 1,000 additional data sets, the 95% confidence interval of the regression coefficients was computed, and variables were removed from the model if zero was included in the confidence interval. Models were only considered further if all variables withstood bootstrapping validation and were significant at *P* ≤ 0.05. If the intercept did not significantly differ from zero, it was also not included in the model. The results were visualized using GraphPad Prism (version 7.0; GraphPad Software Inc., Boston, MA, USA).

Data from all 508 animals in the trial were evaluated with two-way ANOVA by using the following model:


$$\begin{array}{l} y_{ijk}=\mu +\alpha_{i}+\beta_{j}+{\left(\alpha \beta \;\right)}_{ij}+\gamma_{k}+e_{ijk} \end{array}$$ (7)

where *y*_*ijk*_ is the dependent trait, µ is the overall mean, α_*i*_ is the fixed effect of *i*th period, β_*j*_ is the fixed effect of *j*th sex, αβ_*ij*_ is the interaction between *i*th period and *j*th sex, γ_*k*_ is the random effect of *k*th pig, and e_*ijk*_ is the residual error. For ANOVA, Tukey’s adjustment was performed because the number of observations differed between periods. To illustrate the variation, both standard deviation (SD) and % coefficient of variation (CV) were calculated. Pearson correlation coefficients were calculated using the CORR procedure in SAS.

## Results

**Figure 1.**
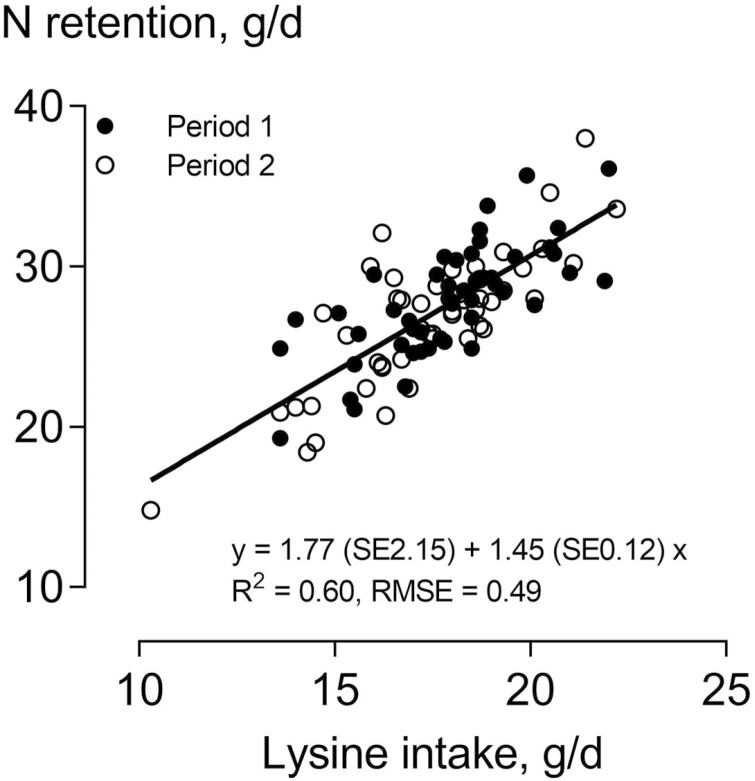
Relationship between nitrogen (N) retention and Lys intake of growing barrows in the N metabolism trial. The estimates of slopes and intercepts were not significantly different between the periods and the regression refers to all data from the N metabolism trial.

**Figure 2.**
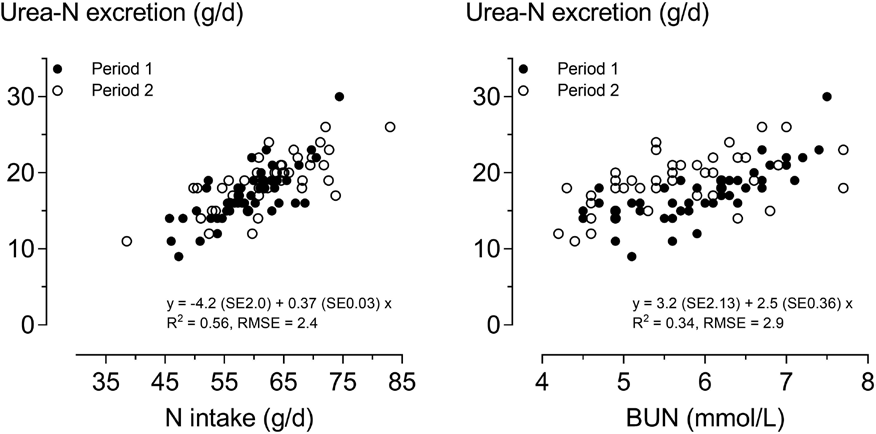
Relationship between urinary urea-nitrogen (N) excretion and N intake (left panel) and blood urea N (BUN) concentration (right panel) of growing barrows in the N metabolism trial. The estimates of slopes of regressions were not significantly different between the periods, and the regressions refer to the pooled data.

In the N metabolism trial, the average BW of the pigs was 40.0 and 59.4 kg in periods 1 and 2, respectively (Table 2). Daily feed and N intake were higher in period 2 than in period 1 (*P* ≤ 0.001). Likewise, N excretion with feces and urine was higher in period 2 than in period 1 (*P* ≤ 0.001), but the ratio of urinary N to fecal N was very similar in both periods and averaged 2.06:1. Daily N retention was on average 27.3 g/d and not significantly different between the two periods. NUE was higher in period 1 than in period 2 (*P* < 0.001); however, the calculated LUE was not, and it was on average 70%. The excretion of urinary urea N was higher in period 2 than in period 1 (*P* < 0.001), and urea N excretion, on average, was 80% of the total urinary N excretion. The excretion of creatinine in urine was higher in period 2 than in period 1 (*P* < 0.001), and the excretion of cortisol in urine was not significantly different. In all studied traits and both periods, the variation among animals was high, as indicated by CV values between 10% and 20% for most of the studied traits. Calculated across all pigs in both periods, an increase in Lys intake of 1 g/d caused an increase in the predicted N retention of 1.45 g/d (Fig. 1). An increase in N intake of 1 g/d caused a mean increase of urea-N excretion of 0.37 g/d (Fig. 2). The concentration of BUN was 5.9 mmol/L in period 1 and 5.7 mmol/L in period 2 (*P* = 0.002; Table 3). The concentration of cortisol in the blood serum was, on average, 22 ng/mL and that of IGF-1 was, on average, 187 ng/mL, and both were not significantly different between the periods.

**Table 2.** Body weight, intake, excretion, and retention of nitrogen (N), N utilization efficiency (NUE), and calculated Lys utilization efficiency (LUE) of barrows in the N metabolism trial (*n* = 48 growing barrows)

Period^1^	1			2			*P*-value
	Mean	SD	%CV	Mean	SD	%CV	
Body weight, kg	40.0	4.47	11.2	59.4	5.25	8.84	<0.001
Feed intake, kg/d	1.69	0.18	10.5	2.21	0.27	12.4	<0.001
N intake, g/d	59.0	6.46	11.0	62.0	7.92	12.8	0.001
Fecal N, g/d	10.1	1.82	18.0	11.6	2.57	22.2	<0.001
Urinary N, g/d	21.0	3.85	18.3	23.7	3.69	15.5	<0.001
N retention, g/d	27.9	3.49	12.5	26.7	4.29	16.1	0.114
NUE, %	47.4	4.13	8.71	43.0	4.05	9.41	<0.001
LUE^2^, %	70.2	6.46	9.20	69.0	6.74	9.76	0.300
Urinary urea N, g/d	17.1	3.58	20.9	18.8	3.43	18.2	<0.001
Urinary creatinine, g/d	1.64	0.24	14.5	2.54	0.41	16.2	<0.001
Urinary cortisol, mg/d	0.20	0.05	23.1	0.21	0.05	25.7	0.965

^1^N metabolism was studied at 13 wk of age (period 1) and 16 wk of age (period 2).

^2^Calculated from measured Lys intake and Lys retention by assuming a value of 0.45 g Lys per 1 g of retained N (GfE, 2008).

**Table 3.** Concentration of urea nitrogen (BUN), serum cortisol (SC), and insulin-like growth factor 1 (IGF-1) in the blood of barrows in the N metabolism trial (*n* = 48 growing barrows)

Period^1^	1			2			*P*-value
	Mean	SD	%CV	Mean	SD	%CV	
BUN, mmol/L	5.94	0.78	13.2	5.70	0.86	15.0	0.022
SC, ng/mL	21.5	8.33	38.8	22.9	11.2	48.8	0.124
IGF-1, ng/mL	204	38.5	18.9	196	37.1	18.9	0.790

^1^N metabolism was studied at 13 wk of age (period 1) and 16 wk of age (period 2).

Among the regressions calculated to estimate NR, the following was chosen to calculate NR from BW and blood metabolites in the entire dataset on the basis of the values of AIC (425), adjusted *R*^2^ (0.72), and root mean square error (RMSE) (1.98) (equation 8).


$$\begin{array}{l} \begin{array}{lllllllll} & NR(g/d)=-32.60-36.01\;BWG\;(kg/d)\; & \;\;\;\;\;\;\;\;\;\;\;\;\;\;\;\;\;\;\;\;\;\;\;\;\;\;\;\;\;\;\;+1.568\;NI\;(g/d)+4.855\;BUN\;(mmol/L)\; & \;\;\;\;\;\;\;\;\;\;\;\;\;\;\;\;\;\;\;\;\;\;\;\;\;\;\;\;\;\;\;+1.169\;IBW\;(kg)*BWG\;(kg/d)\; & \;\;\;\;\;\;\;\;\;\;\;\;\;\;\;\;\;\;\;\;\;\;\;\;\;\;\;\;\;\;\;-0.561\;IBW\;(kg/d)*DMI\;(kg/d)\; & \;\;\;\;\;\;\;\;\;\;\;\;\;\;\;\;\;\;\;\;\;\;\;\;\;\;\;\;\;\;\;+2.125\;DMI\;(kg/d)*BUN\;(mmol/L)\; & \;\;\;\;\;\;\;\;\;\;\;\;\;\;\;\;\;\;\;\;\;\;\;\;\;\;\;\;\;\;\;-0.072\;DMI\;(kg/d)*SC\;(ng/mL)\; & \;\;\;\;\;\;\;\;\;\;\;\;\;\;\;\;\;\;\;\;\;\;\;\;\;\;\;\;\;\;\;-0.154\;NI\;(g/d)*BUN\;(mmol/L)\; & \;\;\;\;\;\;\;\;\;\;\;\;\;\;\;\;\;\;\;\;\;\;\;\;\;\;\;\;\;\;\;+0.001\;SC\;(ng/mL)*IGF\;1\;(ng/mL) \end{array} \end{array}$$ (8)

where BWG is the body weight gain, NI is the nitrogen intake, BUN is the blood urea nitrogen, IBW is the initial body weight, DMI is the dry matter intake, SC is the serum cortisol, and IGF-1 is the insulin-like growth factor 1. Other predicted equations that used less predictor variables are presented in Supplementary Table S1.

**Figure 3.**
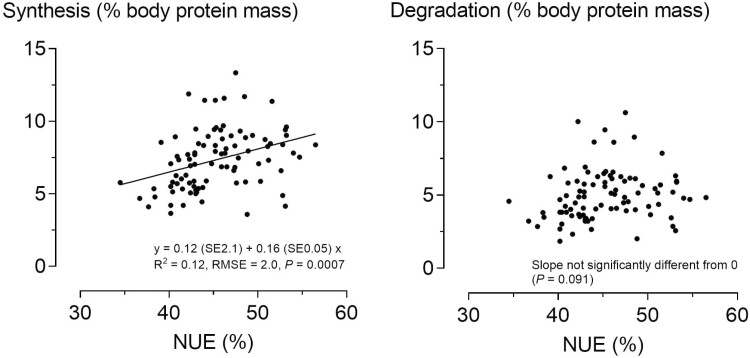
Relationship between nitrogen utilization efficiency (NUE) and fractional synthesis (left panel) and degradation (right panel) of protein in growing barrows (90 measurements in the two periods of the N metabolism trial).

The cumulative ^15^N excretion in urine determined over time in the first two collection periods reached 95% of the estimated plateau after 58 and 65 h in periods 1 and 2, respectively (Supplementary Fig. S1). All other results were based on ^15^N measurement in the urine collected for a total of 84 h. From the administered amount of ^15^N, 20% and 22% were recovered in the urine during periods 1 and 2, respectively (*P* = 0.001) (Table 4). The values of body protein turnover, synthesis, and degradation were not significantly different between the periods. Correlations between the values for each pig in both periods were significant (*P* ≤ 0.02) for protein turnover (0.61), synthesis (0.50), and degradation (0.51). Based on the amount of protein synthesized, the pigs retained an average proportion of 33% during both periods. The NUE values were positively correlated with the rate of protein synthesis (*P* < 0.001) but not with the rate of protein degradation (Fig. 3).

**Table 4.** Amounts of ^15^N administered and recovered in the urine and calculated protein turnover data of barrows in the N metabolism trial

Period^1^	1 (*n* = 48 animals)			2 (*n* = 42 animals)^2^			*P*-value
	Mean	SD	%CV	Mean	SD	%CV	
^15^Nʹ administration, mg	42.0	4.91	11.7	63.7	6.97	10.9	<0.001
^15^Nʹ excretion in urine^3^, mg	8.37	1.93	23.0	14.4	3.42	23.8	<0.001
^15^Nʹ excretion in urine, % of administration	19.8	3.17	16.0	22.4	4.08	18.2	0.001
Protein turnover, g/d	669	104	15.6	668	136	20.3	0.581
Protein synthesis, g/d	538	96.3	17.9	521	130	24.9	0.372
Protein degradation, g/d	364	97.5	26.8	357	125	34.9	0.824
Protein retention/synthesis, %	33.2	6.20	18.7	32.7	7.31	22.3	0.642

^1^N metabolism was studied at 13 wk of age (period 1) and 16 wk of age (period 2).

^2^Administration of the marker failed in 6 animals in period 2.

^3^Excretion in the 84-h period following ^15^N administration.

**Figure 4.**
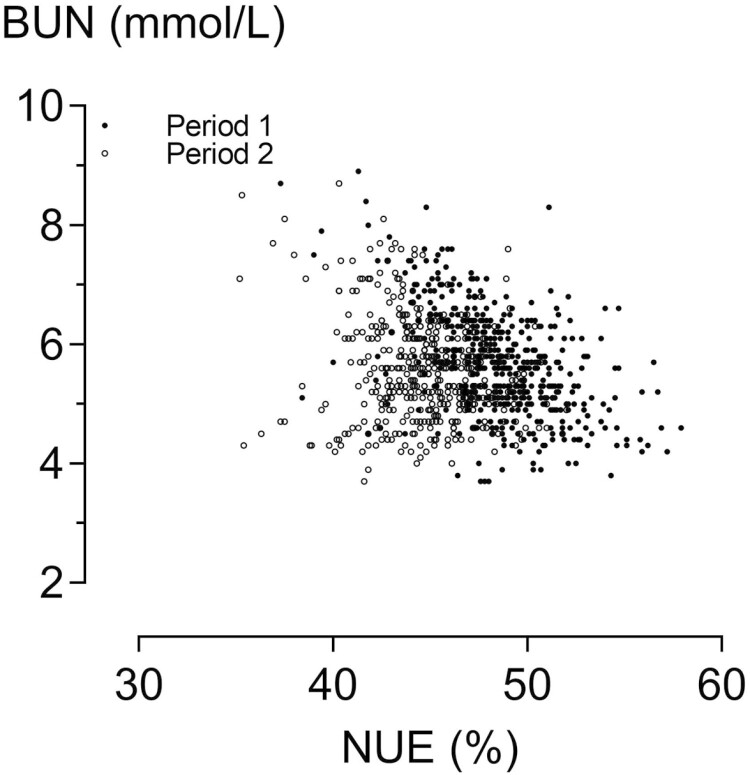
Relationship between nitrogen utilization efficiency (NUE) and blood urea nitrogen (BUN) concentration in growing pigs (data from all 508 pigs of the study). Pearson correlation coefficient was significant in period 1 (−0.50) and period 2 (−0.15).

When evaluating the data of all 508 animals, BWG, DMI, N retention, NUE, and LUE were significantly affected by the period × sex interaction (Table 5). The BWG was, on average, 0.94 kg/day and N retention was, on average, 31.4 g/day, and both were higher in barrows than gilts in period 2 but not in period 1 (*P* < 0.001 for the interaction). The DMI was higher in barrows than in gilts in both periods, but to a greater extent in period 2 than in period 1 (*P* < 0.001). The calculated NUE was, on average, 47%, which was lower in barrows than in gilts in both periods but to a greater extent in period 2 than in period 1 (*P* < 0.001). The calculated LUE was, on average, 71%, which was ­significantly lower in barrows than in gilts in period 2, but not in period 1. The BUN concentration was higher in barrows than in gilts (*P* < 0.001) and higher in period 1 than in period 2 (*P* = 0.006). The BUN concentration was negatively correlated (*P* < 0.05) with NUE in both periods (Figure 4). Blood concentrations of SC were higher in barrows than in gilts to a greater extent in period 2 than in period 1, causing a significant interaction (*P* < 0.001). In contrast, IGF-1 concentration was lower in barrows than in gilts, but this difference was greater in period 2 than in period 1 (*P* < 0.001 for the interaction effect).

**Table 5.** Body weight and gain (BWG), intake of dry matter (DMI), nitrogen (N), and calculated N retention (NR), utilization efficiency (NUE), and lysine utilization efficiency (LUE) of all pigs in the trial (*n* = 508 animals in period 1 and *n* = 458 animals in period 2)^1^

Period	1				2				SEM	*P*-value		
	Gilts		Barrows		Gilts		Barrows			Period	Sex	Period × sex
	Mean	%CV	Mean	%CV	Mean	%CV	Mean	%CV				
BW, kg	40.2^c^	11.6	40.8^c^	11.9	59.5^b^	11.3	61.4^a^	11.7	0.76	<0.001	0.135	<0.001
BWG, kg/d	0.89^c^	13.0	0.91^bc^	15.3	0.93^b^	15.7	1.01^a^	16.3	0.02	<0.001	<0.001	<0.001
DMI, kg/d	1.83^d^	13.5	1.90^c^	13.7	2.41^b^	14.5	2.61^a^	14.0	0.04	<0.001	<0.001	<0.001
N intake, g/d	62.9^d^	13.6	65.1^c^	13.5	68.1^b^	15.0	73.7^a^	15.2	1.25	<0.001	<0.001	<0.001
NR^2^, g/d	30.8^b^	14.9	31.4^ab^	14.4	30.8^b^	17.3	32.4^a^	14.9	0.62	0.133	0.008	0.003
NUE, %	49.0^a^	5.76	48.3^b^	6.23	45.1^c^	5.76	44.0^d^	5.86	0.36	<0.001	<0.001	<0.001
LUE^3^, %	71.5^b^	7.03	70.6^b^	7.21	72.6^a^	7.72	71.0^b^	7.33	0.67	0.006	0.004	<0.001
BUN, mmol/L	5.52^b^	14.9	5.84^a^	15.1	5.34^c^	14.0	5.87^a^	14.9	0.11	0.006	<0.001	0.267
SC, ng/mL	24.0^a^	33.3	25.7^a^	36.3	19.8^c^	38.3	22.0^b^	36.8	1.07	<0.001	0.001	<0.001
IGF-1, ng/mL	213^b^	20.2	202^c^	20.7	223^a^	17.5	196^c^	18.1	5.16	<0.001	<0.001	<0.001

^1^The data collection was not possible for all pigs during the lockdown imposed during Covid-19 pandemic.

^2^Calculated by use of equation (8).

^3^Calculated by use of equation (2).

^a–d^Means in a row not sharing a common superscript letter differ significantly.

## Discussion

### Nitrogen and lysine utilization efficiency

The average daily NR of pigs in both periods was 27.3 g/d in the N metabolism trial and 31.4 g/d in the entire trial. This is at the lower level of the range of data determined in previous N metabolism trials using diets based on wheat, barley, and soybean meal and with ad libitum-fed pigs of similar BW, as in the present study (29 to 33 g/d) (Zervas and Zijlstra, 2002a; Hansen et al., 2006; Barea et al., 2010; Renaudeau et al., 2013). The slightly lower NR when compared with other studies may reflect the marginal Lys concentration in the feed chosen in the present study. On average, daily NR increased by 1.45 g with each 1 g incremental Lys intake (Figure 1). In the study by Moehn et al. (2004), the supply of true digestible Lys to pigs was increased from 60% to 90% of the amount needed for maximum protein deposition. By recalculating the data from this study, the N retention of the pigs increased by 1.44 g with each 1 g of incremental true digestible Lys intake. By consideration of the differences in the approaches of Moehn et al. (2004) and the present study, this can be considered a good agreement of the values. The Lys concentration of body protein in pigs showed some variation among the studies reviewed by Susenbeth (1995), but with reference to gained body protein, the Lys concentration was consistently reported to be 7.2%, without an effect of BW. Considering this value, the efficiency of Lys utilization, calculated from the slope of the regression in Figure 1, was 65%. The same value was calculated by Susenbeth (1995) from N balance studies with pigs, but the author mentioned that this value may represent an overestimation because gaseous N losses can occur in metabolism studies. A slight error of the N balance data might also be related to losses of N with skin and hair (NRC, 2012). Möhn et al. (2000) used diets with true digestible Lys concentrations at 70% and 90% of the requirement of pigs for high protein gain and conducted slaughter and balance studies. They concluded from their results that the marginal efficiency of true digestible Lys utilization for protein gain was 75%. Taken that the estimate of the present study (65%) is based on total Lys intake and that the Lys digestibility of a cereal grain-soybean meal-diet is ~85%, this is considered a good agreement of values. Susenbeth (1995) concluded from a comprehensive data analysis that, when Lys is the first limiting factor, any Lys intake leads to the same protein retention, independent of the age, breed, and sex of the pig. According to the GfE (2008) recommendations, the efficiency of Lys utilization for protein gain in growing pigs is 63%, but this refers to prececal digestible Lys intake. The NRC (2012) assumes that the efficiency of utilization of prececal digestible Lys for protein gain decreases from 68% at 20 kg BW to 57% at 120 kg BW. Taken together, the value of 65% estimated in the present study for the efficiency of total Lys intake is in the upper range of data from other reports and may reflect that the potential for protein gain was reached at a marginal level of Lys in the diet. In contrast to Lys, the supply of other AA exceeded the requirement of the pigs. Because the diets of the present study were not supplemented with free AA, the CP concentration was higher compared to industry-type diets that include AA in free form. Therefore, the level of NUE might be higher than determined in the present study when less CP is used in the feed. Because Lys limited N retention, such CP effect is not likely to occur for the LUE values and for the variation among the pigs that was found in NUE and LUE values.

The CV of NUE and LUE in the N metabolism trial was 9% and it was slightly lower when calculated for all pigs (5.8% to 7.7%), indicating a marked variation among the pigs in the study. Overall, whole-body protein synthesis in pigs in the N metabolism trial was three-fold higher than protein retention. Protein degradation in the magnitude of two-thirds of protein synthesis contributed to AA catabolism and N excretion in the urine. While protein synthesis was significantly related to NUE, protein degradation was not (Fig. 3), indicating that metabolic regulation toward high protein synthesis may lead to higher NUE. The moderate positive Pearson correlation coefficients of IGF-1 with NR (0.20), NUE (0.20), and LUE (0.22) in the N metabolism trial and IGF-1 with NUE for all pigs in periods 1 (0.63) and 2 (0.36) indicated that IGF-1 blood concentrations were related to protein retention and NUE, which is consistent with preliminary studies (Taylor et al., 1992; Whang et al., 2003). IGF-1 is considered to upregulate protein synthesis (Evers, 1989; Zhang et al., 2008), and in the present study a positive Pearson correlation was observed between IGF-1 blood concentration, protein synthesis (0.21), and protein retention (0.20), suggesting that this contributed to the observed effects on NUE. When piglets were muscle-injected with a growth hormone-releasing gene plasmid, increased plasma IGF-1 and protein synthesis but also protein degradation were found, with greater effects on protein synthesis than protein degradation and resulting in an increased protein gain of the piglets (Zhang et al., 2008). An increase in the CP concentration of the diet from 11.8% to 21.8% led to an increase in both serum IGF-1 and protein gain of pigs that were exposed to a CP depletion period until a CP concentration of about 18% was reached (Whang et al., 2003). It is likely that the effect of IGF-1 on protein synthesis was also an influencing factor on NUE in the pigs of the present study.

The end-product method used in the present study was considered particularly useful to study whole-body protein turnover in human population studies which could help to unravel determinants of individual variation (Duggleby and Waterlow, 2005). These authors compared the end-product method using a single dose of ^15^N glycine with the “gold standard” of using continuous intravenous infusion of labeled leucine for 2 or more hours until a steady state of enrichment has been achieved in plasma leucine. They concluded that both methods have advantages and disadvantages and argued “that the end-product method, applied in a strict and standardized way, provides a reasonable estimate of whole-body protein turnover” (Duggleby and Waterlow, 2005). The end-product method was chosen in the present study because a continuous intravenous infusion of the isotope would not have been feasible with the number of pigs we investigated for the characterization of individual variation of whole-body protein turnover.

### Blood urea nitrogen concentration

This study confirms that urea is the main form of N excreted by pigs. Of the total N excreted, two-thirds was excreted in the urine, and thereof 80% was present in the form of urea. Although the proportion of urea-N was unaffected by the amount of NI, urea-N excretion positively correlated with NI (Figure 2). Every 1 g of additional N intake caused an average increase in urea-N excretion of 0.37 g.

The BUN concentrations in pigs vary markedly (Whang and Easter, 2000). In the present study, the BUN values were the means of three samples taken on three subsequent days at the same time each day to reduce variation. BUN concentrations are affected by the time after feeding; however, the time of peak concentration is affected by the CP concentration in the feed, feeding level, and environmental temperature (Berschauer et al., 1983; Zervas and Zijlstra, 2002b). These studies have suggested the peak BUN concentration at “medium” and “high” CP concentration of the feed to occur at 4 to 6 h postfeeding, which guided us to obtain blood 5 h postfeeding in the present study. Nevertheless, the calculated CV of the BUN concentration was 14% in the N metabolism trial and at a similar level in both sexes when calculated for all pigs in this study.

The BUN concentration is well known for its association with total urinary N excretion at variable CP concentrations in the feed (Mosenthin et al., 1992; Zervas and Zijlstra, 2002a; Kohn et al., 2005). In the present study, the BUN concentration was also positively correlated with the excretion of urea-N (Figure 2). This suggests that BUN concentration is an indicator of urinary N excretion in pigs, not only when different CP levels in the feed are used but also when all pigs are fed the same diet. Regarding the relationship between BUN and NUE, the results obtained from all the pigs were not fully consistent with those of the N metabolism trial. In all pigs, BUN concentrations were negatively correlated with NUE in both periods (Fig. 4), whereas a negative correlation existed in the N metabolism trial only in period 1 and no correlation existed when both periods were evaluated together. Differences in the number of observational units may have caused these differences between datasets. Other studies have found a stronger relationship and suggested that BUN is a useful indicator for assessing NUE (Berschauer et al., 1983; Whang et al., 2003). However, these studies used different CP concentrations in the feed, and increased AA catabolism increases urea synthesis and circulation (Wilson et al., 1972). In the present study, all pigs were fed the same diet with marginal Lys concentration. Thus, the variation in CP intake resulted from the differences in only feed intake. In the study by Whang and Easter (2000), BUN concentrations were lower when the lean gain of pigs was higher. In this study, a restricted amount of feed was provided after 14 h of fasting, which might have affected the BUN responses when compared with non-fasted pigs. When two distinct pig genotypes were compared in the study by Fabian et al. (2003), BUN concentration was lower in the line selected for lean growth efficiency than in the control line when the same feed was provided. However, N digestibility was also lower in the selected line, suggesting that fewer amino acids entered the blood pool and were catabolized, and urea was synthesized. In the study by Zhang et al. (2008), NUE increased and BUN decreased in piglets injected with a growth hormone-releasing gene plasmid. Thus, for different purposes, BUN was shown to be a useful indicator of NUE, even at the same CP content in the feed, which was also indicated by the results from all pigs in the present study.

Consistent with the results of previous studies (Chen et al., 1999; Whang and Easter, 2000), the BUN concentrations in the present study were higher in barrows than in gilts. Feed and CP intake were higher in barrows than in gilts, which probably caused higher urea synthesis. The feed intake of barrows was also higher in the study by Chen et al. (1999), whereas no feed intake was reported by Whang and Easter (2000).

In conclusion, under conditions of marginal Lys supply, variation in NUE in a pig population is high and may be estimated by the BUN concentration. Thus, BUN may be considered as useful in phenotyping NUE of individual pigs in a large population. The rate of whole-body protein synthesis, rather than protein degradation, seemed to affect NUE under the conditions of this study.

## Supplementary Material

skad335_suppl_Supplementary_Figures_S1_Tables_S1

## Abbreviations:

AIC: Akaike information criterion
BUN: blood urea nitrogen
BW: body weight
BWG: body weight gain
CP: crude protein
DM: dry matter
DMI: dry matter intake
IGF-1: insulin-like growth factor 1
LUE: lysine utilization efficiency
N: nitrogen
NI: nitrogen intake
NR: nitrogen retention
NUE: nitrogen utilization efficiency
RMSE: root mean square error
SD: standard deviation
SC: serum cortisol
CV: coefficient of variation
