# Supplementary material for: Nitrogen and lysine utilization efficiencies, protein turnover, and blood urea concentrations in crossbred grower pigs at marginal dietary lysine concentration
Source: J Anim Sci. 2023 Sep 29;101:skad335. doi: 10.1093/jas/skad335 (PMC10583982; doi:10.1093/jas/skad335)
Supplement: skad335_suppl_Supplementary_Figures_S1_Tables_S1 [file skad335_suppl_supplementary_figures_s1_tables_s1.docx]

**Supplemental file to the manuscript**

**Nitrogen and lysine utilization efficiencies, protein turnover, and blood urea concentrations in crossbred grower pigs at marginal dietary lysine concentration**

by Daniel Berghaus, Eva Haese, Ramona Weishaar, Naomi Sarpong, Alina Kurz, Jana Seifert, Amélia Camarinha Silva, Jörn Bennewitz, Thilo Chillon, Volker Stefanski, and Markus Rodehutscord

**Fig. S1:** Cumulative excretion of ^15^N in the urine of pigs following a single oral administration of ^15^N glycine at a dosage of 1.18 mg ^15^N/kg BW (mean and SE; n = 8 per period).

**Table S1.** Additional equations for estimating nitrogen retention (g/d) of growing pigs fed diets with marginal lysine supply based on N balance data and blood metabolites

| Eq. | Variable | b | SEb | *p*-Value | AIC | adj. R^2^ | RMSE |
| --- | --- | --- | --- | --- | --- | --- | --- |
| A1 |  |  |  |  | 408 | 0.73 | 2.01 |
|  | Intercept | -37.82 | 5.941 | <0.001 |  |  |  |
|  | BW | -0.147 | 0.019 | <0.001 |  |  |  |
|  | NI | 0.579 | 0.039 | <0.001 |  |  |  |
|  | CP ATTD | 0.564 | 0.073 | <0.001 |  |  |  |
|  | BUN | -1.543 | 0.352 | <0.001 |  |  |  |
| A2 |  |  |  |  | 438 | 0.67 | 2.27 |
|  | BW | -0.137 | 0.022 | <0.001 |  |  |  |
|  | NI | 0.479 | 0.026 | <0.001 |  |  |  |
|  | SC | 0.055 | 0.024 | 0.024 |  |  |  |
|  | IGF-1 | 0.018 | 0.006 | 0.001 |  |  |  |
| A3 |  |  |  |  | 449 | 0.62 | 2.41 |
|  | Intercept | 5.067 | 2.153 | 0.021 |  |  |  |
|  | BW | -0.134 | 0.024 | <0.001 |  |  |  |
|  | NI | 0.478 | 0.039 | <0.001 |  |  |  |

Regression coefficients (b) and standard errors (SEb) were obtained using multiple regression. AIC = Akaike information criterion; BW = body weight, kg; NI = nitrogen intake, g/d; CP ATTD = apparent total tract crude protein digestibility, %; BUN = blood urea nitrogen, mmol/L; SC = serum cortisol, ng/mL; IGF-1 = insulin-like growth factor 1, ng/mL; RMSE = root mean square error.
